# A Comparative Morphometrical Study of the Pecten Oculi in Different Avian Species

**DOI:** 10.1155/2013/968652

**Published:** 2013-05-22

**Authors:** Mustafa Orhun Dayan, Tugba Ozaydın

**Affiliations:** ^1^Department of Anatomy, Faculty of Veterinary Medicine, University of Selcuk, 42075 Konya, Turkey; ^2^Department of Histology and Embryology, Faculty of Veterinary Medicine, University of Selcuk, 42075 Konya, Turkey

## Abstract

In this study was investigated the structure of pecten oculi in the ostrich, duck, pigeon, turkey, and starling. The pecten oculi of the ostrich was vaned type and made up primary, secondary, and few tertiary lamellae. However, duck, pigeon, turkey and starling had a pleated-type pecten oculi which displayed folded structure. The numbers of pleats of the pectens were 12, 13-14, 21-22, and 17 in duck, pigeon, turkey, and starling, respectively. Light microscopic investigation demonstrated that pecten oculi is basically composed of numerous capillaries, large blood vessels, and pigment cells in all investigating avian species. Capillaries were 20.23, 14.34, 11.78, 12.58, and 12.78 **μ**m in diameter in ostrich, duck, pigeon, turkey, and starling, respectively. The capillaries are surrounded by thick basal membrane, and pigmented cells were observed around the capillaries.

## 1. Introduction

The pecten oculi is a highly vascular and pigmented structure peculiar to the avian eye [1–3]. It is situated over the optic nerve head and projects from the retina into the vitreous body [4, 5]. 

Three morphological types of pecten oculi are recognized as conical, vaned, and pleated types. The conical type is only reported in the brown kiwi (*Apteryx mantelli*); the vaned type is present in ostriches (*Struthio camelus*) and rheas (*Rhea americana*) [6]; and pleated form is widely reported in most birds (neognathae) such as quail [5], black kite [1], galah [7], common buzzard [3], mallard [8], pigeon [4], and jungle crow [9]. The size of the pecten depends on the visual requirements of the bird, so that daily active bird species have a relatively large and highly complex pecten oculi with many folds while night active bird species will have a relatively small and simple pecten oculi [6].

Histologic studies have shown that the pecten oculi consists of blood vessels, extravascular pigmented cells, and superficial covering membrane [1]. Birds have thicker retinas compared to mammals but have not retinal blood vessels [10]. Therefore it has been suggested that the main function of pecten oculi is to supply nutrition to the avascular avian retina [1]. One of functions of pecten is the formation of a blood-retina barrier [11]. The endothelia of the pectinal capillaries are continuous, possessing elaborate tight junctions. Also two barrier-specific proteins, that is, the HT7-antigen and the glucose transporter isoform GluT-1, are expressed by the endothelial cells [12].

Significant variations in the pecten oculi such as type and number of pleats exist within the avian species due to the behavior of birds in relation to their general activity and visual pattern. In the present study we studied the anatomical and histological details of the pecten oculi in the ostrich (Struthioniformes), duck (Anseriformes), pigeon (Columbiformes), turkey (Galliformes), and starling (Passeriformes) which belong to different orders of the avian species.

## 2. Materials and Methods

The eyeballs were obtained from adult ostrich, duck, pigeon, turkey, and starling. Six animals from each species were used in this study. The animals were anaesthetized and decapitated and the eye rapidly enucleated. The eyeballs were cut at the equator, and the posterior half which contained the pecten oculi was photographed using digital camera attached stereomicroscope (Nikon SMZ- 2T, Nikon Corp., Tokyo, Japan). The number of pleats of duck, pigeon, turkey, and starling pecten oculi was counted in stereomicroscope images.

The pecten oculi and its underlying retinal tissue were painstakingly dissected out, then they were fixed in 10% buffered formalin for histologic examination. After the fixation, the samples dehydrated in increasing concentrations of ethanol, cleared with xylene, and embedded in paraffin. The serial sections from the apex to the base of pecten were cut in 6 *μ*m thick, and the sections were stained using Crossman's triple technique [13] and periodic-acid Schiff (PAS) reaction for basement membrane composed of glycoconjugates [14].

All specimens were examined under light microscope (Leica DM-2500 model with DFC-320 camera attachment giving digital images). The diameter of the capillaries and thickness of capillary basement membrane of pecten oculi were measured with IM-50 image analysis program. The parameters were analyzed with one-way ANOVA (SPPSS 9.0; SPSS Inc. Corp., USA). Results were considered significant at *P* < 0.05.

## 3. Results

**Figure 1:**
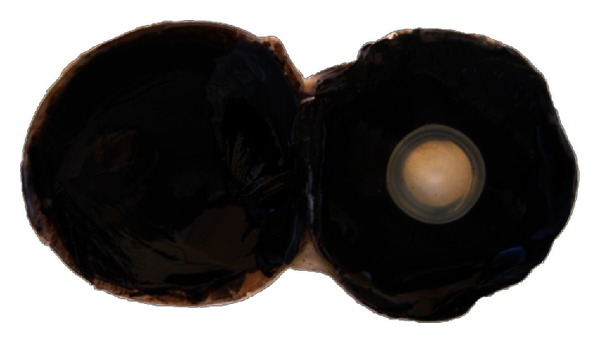
The appearance of pecten oculi of the ostrich.

**Figure 2:**
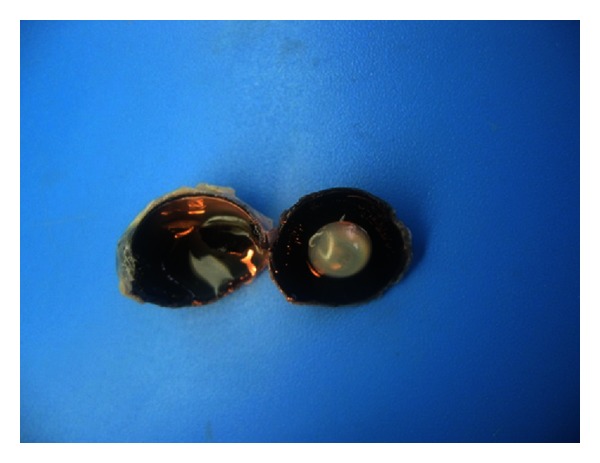
The appearance of pecten oculi of the duck.

The pecten oculi was located over the optic disc and was projected out into the vitreous body. Due to the pigmentation pecten oculi was observed in dark brown to black (Figures 1 and 2).

**Figure 3:**
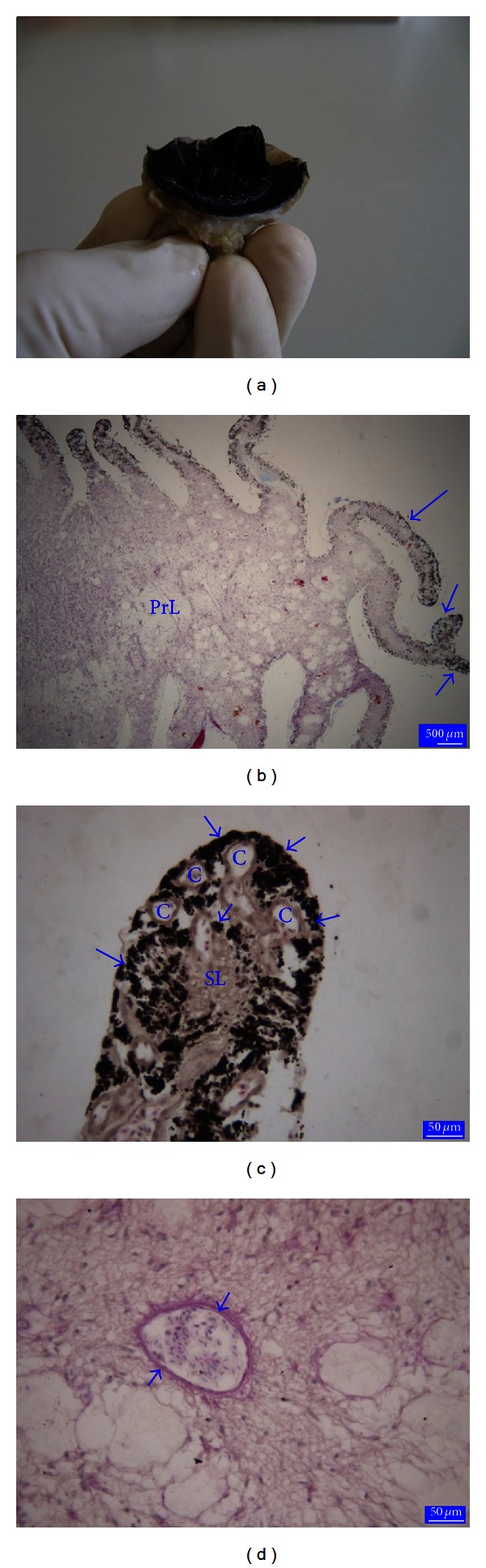
The appearance of pecten oculi of the ostrich (a). Paraffin sections from the ostrich pecten oculi (b, c, and d). PrL: Primary lamellae, large arrows: secondary lamellae, small arrows: tertiary lamellae, Crossmon's trichrome stain (b). SL: secondary lamellae, C: blood capillaries, arrows: pigmentation, Crossmon's trichrome stain (c). Arrows: thick basement membrane, PAS (d).

The pecten oculi of the ostrich was vanned type (Figure 3(a)) and made up primary, secondary, and few tertiary lamellae (Figure 3(b)). Centrally located primary lamellae were the thicker lamellae, and the thinner secondary lamellae were originated from the primary lamellae. The tertiary lamellae arose from the distal part of the secondary lamellae were rarely. In primary lamellae, there were the large blood vessels having thick basement membrane. The blood capillaries were especially located in the distal part of the secondary lamellae (Figure 3(c)). The wall of these capillaries is composed of a single layer of high endothelial cells surrounded by a thick basement membrane (Figure 3(d)). High degree of pigmentation distributed between the capillaries was the most salient feature of the distal part of the secondary lamellae (Figure 3(c)).

**Figure 4:**
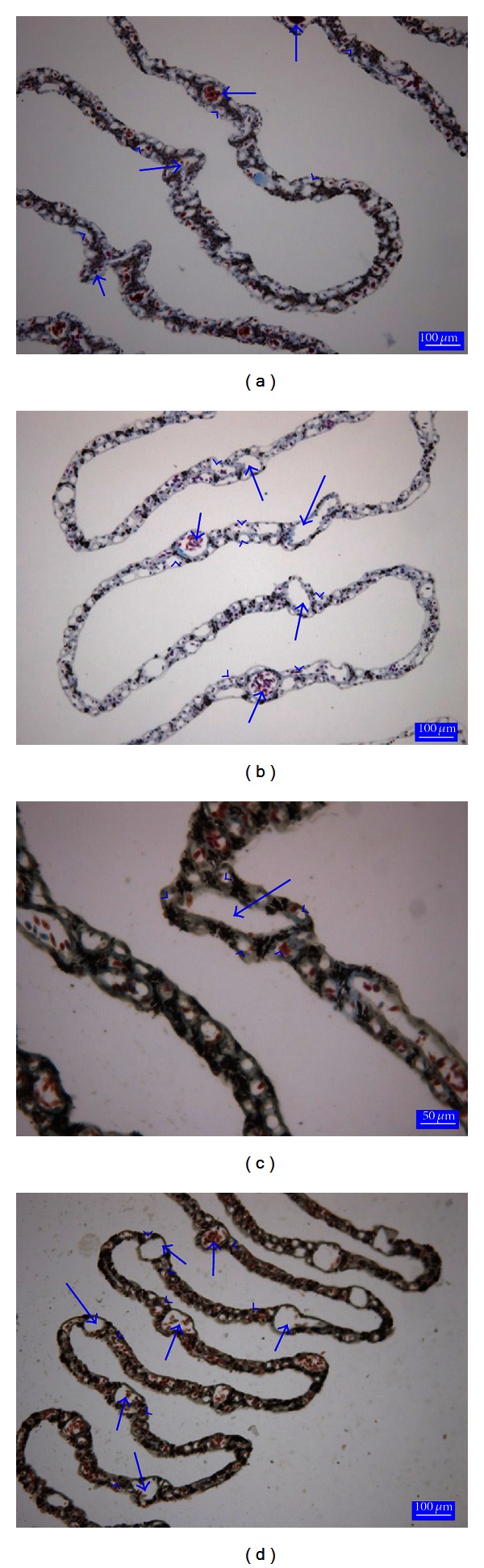
A section from the duck pecten oculi (a); a section from the pigeon pecten oculi (b); a section from the turkey pecten oculi (c); a section from the starling pecten oculi (d); arrows: blood vessels, arrow heads: blood capillaries. Crossmon's trichrome stain.

**Figure 5:**
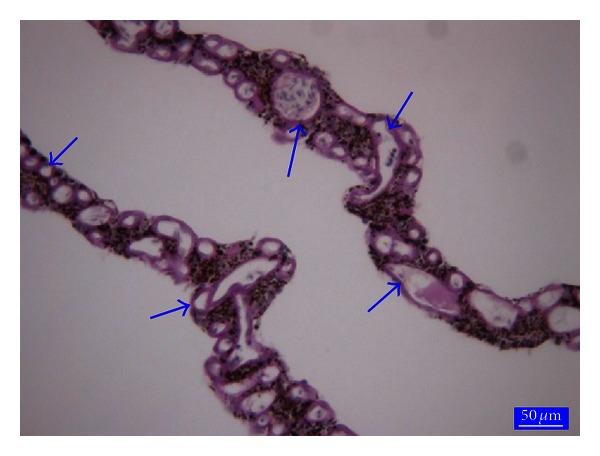
A section from the duck pecten oculi. Arrows: Large blood vessel and many capillaries having thick basement membrane. PAS, bar 30 *μ*m.

In duck, pigeon, turkey, and starling the pecten oculi consists of the several thin folds which confluence each other at the apex were very delicate (Figures 4(a), 4(b), 4(c), and 4(d)). In serial transverse sections it was revealed that each fold has a large blood vessel surrounded by many capillaries having high endothelium and thick basement membrane (Figure 5). The distribution of the pigmentation was similar to ostrich pecten oculi between the capillaries. The number of pleats and the other morphometric parameters of pecten oculi were illustrated in Table 1.

## 4. Discussion

The location of the pecten oculi in all avian species used in this study conformed to that reported in other bird species. We observed that duck, pigeon, turkey, and starling had a pleated-type pecten oculi which displayed folded structure. However the pecten oculi of the ostrich was vaned type and made up primary, secondary, and few tertiary lamellae as cited by Kiama et al. [2]. 

Pleated-type pecten oculi has similar structures in various avian species as in this study. However some considerable variations between the species can be observed in number of the pleats, size, shape and thickness of the capillary basal lamina of the pecten oculi. These variations depend on the diurnal activity and visual requirements of the species. Generally, daily active birds (diurnal) have bigger and more plated pectens compared to night active birds (nocturnal) [7, 15]. In this the study number of pleats of the pectens was 12, 13-14, 21-22, and 17 in duck, pigeon, turkey, and starling, respectively. Previous studies have demonstrated that other diurnal species such as domestic fowl has 16–18 [15], black kite has 12-13 [1, 15], quail has 19 [5], common buzzard has 17-18 [3], jungle crow has 24-25 [9], mallard has 12–14 [8] folds in pectin. However nocturnal birds have small pectens such as barred owl that has 8–10 [16] and spotted eagle owl that has 5-6 [15] folds in pecten. 

Although functional morphology of the pecten oculi correlates with the life-style of the bird [15], histologic findings obtained from this study and previous study [2, 5, 8] in various bird species demonstrated that pecten oculi is basically composed of numerous capillaries, large blood vessels, and pigment cells. High vascular structure and specialized capillary morphology is important characteristic for nutritive function of this organ. The capillaries are surrounded by thick basal membrane in pecten oculi of all investigated species in this study as described in pervious study [4, 7, 8, 17]. It is suggested that thickened basal laminae may support the fragile endothelial cells which have very thin cell bodies and numerous microfolds [7, 17]. 

The pigmented cells are the second prominent cell type of the pecten oculi [18]. Pigmented cells were observed around the capillaries in this study. The close association between the pigmented cells and the capillaries has been also reported in the black kite [1], quail [5], ostrich [2], and jungle crow [9]. It has suggested that pigmented cells provide the structural reinforcement to pecten oculi for keeping it firmly erectile within the gel-like vitreous and also protect the blood vessels against damage from ultraviolet light [1, 7, 17]. In addition the absorption of light by the pigmented cells probably raises the temperature within the pecten and hence the rate of metabolism within it [19].

This research provides data concerning the anatomical and histological characteristics of pecten oculi of different avian species. These findings reveal that pecten oculi in the ostrich was vaned type, and duck, pigeon, turkey, and starling had pleated-type pecten oculi. Histologic structures were quite similar in pecten oculi of all investigated species. 

## Figures and Tables

**Table 1:** The morphometric parameters of pecten oculi in different avian species.

Animals	The numbers of pleats	Diameter of capillaries (*µ*m) (*X* ± Sd)	Thickness of capillary basement membrane (*µ*m) (*X* ± Sd)
Ostrich	—	20.23 ± 2.98^a^	2.67 ± 0.32
Duck	12	14.34 ± 2.56^b^	2.11 ± 0.55
Pigeon	13-14	11.78 ± 2.71^b^	2.24 ± 0.37
Turkey	21–22	12.58 ± 2.67^b^	2.08 ± 0.56
Starling	17	12.78 ± 1.50^b^	2.00 ± 0.32

^a-b^Values within a column with no common superscripts are significantly different (*P* < 0.05).
